# Chicago Medical College

**Published:** 1874-05-01

**Authors:** 


CHICAGO MEDICAL COLLEGE.—At the recent annual meeting of the Trustees of the College Dr. Thos. S. Bond was appointed Professor of Descriptive Anatomy, in the place of Prof. H. W. Boyd, resigned. Prof. H. P. Merriman was transferred to the Chair of Medical Jurisprudence, made vacant by the resignation of Prof. R. J. Patterson. Prof. W. S. Haines was appointed Professor of Organic Chemistry and Toxicology, in the place of Prof. Merriman, transferred. Dr. Charles L. Rutter was appointed Demonstrator of Anatomy, in place of Dr. T. S. Bond, transferred.

The summer course is now in progress, and well attended.

---

OBSTRUCTIONS OF THE RECTUM BY UTERINE TUMORS. — This subject was considered at the Surgical Society of Paris in October.

Fibrous uterine tumors occasion intestinal obstructions more frequently than is generally supposed; they have yielded in some cases to maneuvers made externally to effect the displacement of the tumor. Cases were quoted of obstruction of the intestines by these uterine tumors, and one where death resulted as much from the obstruction as from peritonitis. Intervention by operation is rare, on account of the risk of peritonitis; but M. Gueniot recommends colotomy, if other curative measures fail.

